# Supplementary material for: ESCRT-III activation by parallel action of ESCRT-I/II and ESCRT-0/Bro1 during MVB biogenesis
Source: eLife. 2016 Apr 13;5:e15507. doi: 10.7554/eLife.15507 (PMC4865371; doi:10.7554/eLife.15507)
Supplement: Supplementary file 1. — DOI: http://dx.doi.org/10.7554/eLife.15507.020 [file elife-15507-supp1.docx]

**Supplemental file 1, related to MATERIALS AND METHODS. Plasmids and Yeast Strains Used in This Study**

| **Plasmids for *Saccharomyces cerevisiae* Expression** | | | | | |
| --- | --- | --- | --- | --- | --- |
| **Plasmid** | | **Mutations** | | | **Reference** |
| pRS416 | | N/A | | | (Sikorski and Hieter, 1989) |
| pRS415 | | N/A | | | (Sikorski and Hieter, 1989) |
| pRS414 | | N/A | | | (Sikorski and Hieter, 1989) |
| pRS416-*VPS20* | | Wild-type | | | this study |
| pRS416-*vps20^1-197^-snf7^211-240^* | | Vps20^1-197^-Snf7^211-240^ | | | this study |
| pRS416-*vps20^1-172^-snf7^181-240^* | | Vps20^1-172^-Snf7^181-240^ | | | this study |
| pRS416-*vps20^1-147^-snf7^150-240^* | | Vps20^1-147^-Snf7^150-240^ | | | this study |
| pRS416-*vps20^1-118^-snf7^120-240^* | | Vps20^1-118^-Snf7^120-240^ | | | this study |
| pRS416-*vps20^1-105^-snf7^107-240^* | | Vps20^1-105^-Snf7^107-240^ | | | this study |
| pRS416-*vps20^1-59^-snf7^61-240^* | | Vps20^1-59^-Snf7^61-240^ | | | this study |
| pRS416-*vps20^1-5^-snf7^11-240^* | | Vps20^1-5^-Snf7^11-240^ | | | (Buchkovich et al., 2013) |
| pRS416-*SNF7* | | Wild-type | | | (Henne et al., 2012) |
| pRS416-*snf7^Q90L^* | | Q90L | | | this study |
| pRS416-*snf7^N100I^* | | N100I | | | this study |
| pRS416-*snf7^R52E^* | | R52E | | | (Henne et al., 2012) |
| pRS416-*snf7^R52E Q90L^* | | R52E Q90L | | | this study |
| pRS416-*snf7^R52E Q90L N100I^* | | R52E Q90L N100I | | | this study |
| pRS416-*snf7*** | | Vps20^1-5^-Snf7^11-240^ ^R52E Q90L^ | | | this study |
| pRS416-*snf7**** | | Vps20^1-5^-Snf7^11-240^ ^R52E Q90L N100I^ | | | this study |
| pRS415-*snf7*** | | Vps20^1-5^-Snf7^11-240^ ^R52E Q90L^ | | | this study |
| pRS415-*snf7**** | | Vps20^1-5^-Snf7^11-240^ ^R52E Q90L N100I^ | | | this study |
| pRS414-*snf7*** | | Vps20^1-5^-Snf7^11-240^ ^R52E Q90L^ | | | this study |
| pRS414-*snf7**** | | Vps20^1-5^-Snf7^11-240^ ^R52E Q90L N100I^ | | | this study |
| pRS426-*GFP-CPS1* | | GFP | | | (Odorizzi et al., 1998) |
| pRS414-*vam7^tsf^* | | L134P L287P | | | (Sato et al., 1998) |
| pRS415-*VPS20* | | Wild-type | | | (Buchkovich et al., 2013) |
| pRS414-*SNF7* | | Wild-type | | | (Tang et al., 2015) |
| pRS414-*VPS24* | | Wild-type | | | (Buchkovich et al., 2013) |
| pRS415-*VPS2* | | Wild-type | | | (Buchkovich et al., 2013) |
| pRS416-*VPS4* | | Wild-type | | | this study |
| pRS416-*VPS36-MYC* | | Wild-type | | | (Hierro et al., 2004) |
| pRS416-*VPS22-HA* | | Wild-type | | | (Hierro et al., 2004) |
| pRS415-*VPS25* | | Wild-type | | | this study |
| pRS416-*VPS23* | | Wild-type | | | this study |
| pRS415-*VPS23* | | Wild-type | | | this study |
| pRS416-*VPS28* | | Wild-type | | | this study |
| pRS416-*VPS37* | | Wild-type | | | this study |
| pRS416-*VPS27* | | Wild-type | | | this study |
| pRS416-*HSE1* | | Wild-type | | | this study |
| pRS416-*vps27^UIM^* | | S270D S313D | | | this study |
| pRS416-*BRO1* | | Wild-type | | | (Odorizzi et al., 2003) |
| pRS416-*bro1^UBD^* | | I377R L386R | | | this study |
| pRS415-*snf7*** ^BRO1^* | | Vps20^1-5^-Snf7^11-240^ ^R52E Q90L N100I L231K L234K^ | | | this study |
| pRS416-*bro1^SNF7^* | | I144D L336D | | | this study |
| pRS415-*vps20^1-200^* | | Vps20^1-200^ | | | this study |
| pRS415-*vps20^1-172^* | | Vps20^1-172^ | | | this study |
| pRS415-*vps20^1-147^* | | Vps20^1-147^ | | | this study |
| pRS416-*vps20^1-59^-snf7^61-240 Q90L^* | | Vps20^1-59^-Snf7^61-240 Q90L^ | | | this study |
| pRS416-*snf7^7mut^* | | T20D V24L R27K I30K N31D L33I S34H | | | this study |
| pRS416-*snf7^7mut R52E Q90L^* | | T20D V24L R27K I30K N31D L33I S34H R52E Q90L | | | this study |
| pRS416-*snf7^7mut R52E Q90L N100I^* | | T20D V24L R27K I30K N31D L33I S34H R52E Q90L N100I | | | this study |
| pRS416-*snf7** ^7mut^* | | Vps20^1-5^-Snf7^11-240^ ^R52E Q90L^ T20D V24L R27K I30K N31D L33I S34H | | | this study |
| pRS416-*snf7*** ^7mut^* | | Vps20^1-5^-Snf7^11-240^ ^R52E Q90L N100I^ T20D V24L R27K I30K N31D L33I S34H | | | this study |
| pRS416-*vps36^arm^-MYC* | | D548R | | | this study |
| pRS416-*vps22^arm^-HA* | | D214A | | | (Hierro et al., 2004) |
| pRS416-*vps25^arm^-FLAG* | | R83D | | | (Hierro et al., 2004) |
| pRS416-*VPH1-GFP* | | GFP | | | this study |
| pRS426-*GFP-CPS1^K8R K12R^* | | GFP, K8R K12R | | | this study |
|  | |  | | |  |
| **Plasmids for *Escherichia coli* Expression for Protein Purification** | | | | | |
| **Plasmid** | | **Mutations** | | **Reference** | |
| pET23d-*SNF7* | | Wild-type | | (Henne et al., 2012) | |
| pET23d-*snf7*^R52E^ | | R52E | | (Henne et al., 2012) | |
| pET23d-*snf7*^Q90L^ | | Q90L | | this study | |
| pET23d-*snf7^α1-α4^* | | Snf7^10-150^ | | (Buchkovich et al., 2013) | |
| pET23d-*snf7^α1-α4 Q90L^* | | Snf7^10-150 Q90L^ | | this study | |
| pET23d-*snf7^α1-α4 N100I^* | | Snf7^10-150 N100I^ | | this study | |
| pET23d-*BRO1* | | Wild-type | | this study | |
| pET23d-*snf7*^1-225^ | | Snf7^1-225^ | | this study | |
|  | |  | |  | |
| **Yeast Strains Used in This Study** | | | | | |
| **Strain** | | | **Genotype** | **Reference** | |
| SEY6210 | *Mat α, leu2-3, 2-112, ura3-52, his3-*Δ*200, trp1-*Δ*901, lys2-801, suc2-*Δ*9* | | | (Robinson et al., 1988) | |
| SEY6210.1 | *Mat a, leu2-3, 2-112, ura3-52, his3-*Δ*200, trp1-*Δ*901, lys2-801, suc2-*Δ*9* | | | (Robinson et al., 1988) | |
| NBY42 | SEY6210.1; *vps20*Δ*::HIS3; MUP1-PHLOURIN::KAN* | | | (Buchkovich et al., 2013) | |
| MBY25 | SEY6210.1; *vps20*Δ*::HIS3* | | | (Babst et al., 2002) | |
| NBY44 | SEY6210.1; *snf7*Δ*::HIS3; MUP1-PHLOURIN::KAN* | | | (Henne et al., 2012) | |
| NBY80 | SEY6210.1; *vps20*Δ*::HIS3 vam7*Δ*::HIS3* | | | this study | |
| NBY47 | SEY6210.1; *vps24*Δ*::HIS3; MUP1-PHLOURIN::KAN* | | | (Buchkovich et al., 2013) | |
| NBY69 | SEY6210.1; *vps2*Δ*::HIS3; MUP1-PHLOURIN::KAN* | | | (Buchkovich et al., 2013) | |
| NBY59 | SEY6210.1; *vps4*Δ*::HIS3; MUP1-PHLOURIN::KAN* | | | this study | |
| STY55 | SEY6210.1; *vps36*Δ*::TRP1; MUP1-PHLOURIN::KAN* | | | this study | |
| STY56 | SEY6210.1; *vps22*Δ*::TRP1; MUP1-PHLOURIN::KAN* | | | this study | |
| STY51 | SEY6210; *vps25*Δ*::HIS3; MUP1-PHLOURIN::KAN* | | | this study | |
| STY77 | SEY6210; *vps36*Δ*::TRP1; vps25*Δ*::HIS3; MUP1-PHLOURIN::KAN* | | | this study | |
| NBY63 | SEY6210.1; *vps23*Δ*::HIS3; MUP1-PHLOURIN::KAN* | | | this study | |
| SBY1 | SEY6210.1; *vps28*Δ*::TRP1; MUP1-PHLOURIN::KAN* | | | this study | |
| STY76 | SEY6210.1; *vps37*Δ*::TRP1; MUP1-PHLOURIN::KAN* | | | this study | |
| STY64 | SEY6210; *vps23*Δ*::TRP1; vps25*Δ*::HIS3; MUP1-PHLOURIN::KAN* | | | this study | |
| STY31 | SEY6210.1; *vps27*Δ*::HIS3; MUP1-PHLOURIN::KAN* | | | this study | |
| STY57 | SEY6210.1; *vps27*Δ*::TRP1; vps20*Δ*::HIS3; MUP1-PHLOURIN::KAN* | | | this study | |
| STY63 | SEY6210.1; *vps27*Δ*::TRP1; vps25*Δ*::HIS3; MUP1-PHLOURIN::KAN* | | | this study | |
| STY62 | SEY6210.1; *vps27*Δ*::TRP1; vps23*Δ*::HIS3; MUP1-PHLOURIN::KAN* | | | this study | |
| YAY16 | SEY6210; *bro1*Δ*::HIS3; MUP1-PHLOURIN::KAN* | | | this study | |
| YAY20 | SEY6210; *bro1*Δ*::HIS3; vps20*Δ*::HIS3; MUP1-PHLOURIN::KAN* | | | this study | |
| STY65 | SEY6210; *bro1*Δ*::HIS3; vps25*Δ*::TRP1; MUP1-PHLOURIN::KAN* | | | this study | |
| STY66 | SEY6210; *bro1*Δ*::HIS3; vps23*Δ*::TRP1; MUP1-PHLOURIN::KAN* | | | this study | |
| DTY36 | SEY6210.1; *vps20*Δ*::HIS3; SNF7-GFP::HIS3* | | | (Teis et al., 2008) | |
| NBY75 | SEY6210.1; *vps20*Δ*::HIS3; snf7*Δ*::HIS3; MUP1-PHLOURIN::KAN* | | | this study | |
| STY59 | SEY6210.1; *vps24*Δ*::TRP1; vps20*Δ*::HIS3; MUP1-PHLUORIN::KAN* | | | this study | |
| STY60 | SEY6210.1; *vps2*Δ*::TRP1; vps20*Δ*::HIS3; MUP1-PHLUORIN::KAN* | | | this study | |
| STY61 | SEY6210.1; *vps4*Δ*::TRP1; vps20*Δ*::HIS3; MUP1-PHLUORIN::KAN* | | | this study | |
| STY52 | SEY6210.1; *vps36*Δ*::TRP1; vps20*Δ*::HIS3; MUP1-PHLUORIN::KAN* | | | this study | |
| STY53 | SEY6210.1; *vps22*Δ*::TRP1; vps20*Δ*::HIS3; MUP1-PHLUORIN::KAN* | | | this study | |
| STY54 | SEY6210.1; *vps25*Δ*::TRP1; vps20*Δ*::HIS3; MUP1-PHLUORIN::KAN* | | | this study | |
| STY58 | SEY6210.1; *vps23*Δ*::TRP1; vps20*Δ*::HIS3; MUP1-PHLUORIN::KAN* | | | this study | |
| STY72 | SEY6210.1; *vps28*Δ*::TRP1; vps20*Δ*::HIS3; MUP1-PHLUORIN::KAN* | | | this study | |
| STY73 | SEY6210.1; *vps37*Δ*::TRP1; vps20*Δ*::HIS3; MUP1-PHLUORIN::KAN* | | | this study | |
| NBY55 | SEY6210.1; *vps23*Δ*::HIS3; vam7*Δ*::HIS3* | | | this study | |
| STY78 | SEY6210.1; *vps20*Δ*::HIS3; hse1*Δ*::TRP1; MUP1-PHLUORIN::KAN* | | | this study | |
| STY79 | SEY6210; *vps25*Δ*::HIS3; hse1*Δ*::TRP1; MUP1-PHLUORIN::KAN* | | | this study | |
| STY80 | SEY6210.1; *vps23*Δ*::HIS3; hse1*Δ*::TRP1; MUP1-PHLUORIN::KAN* | | | this study | |

**REFERENCE**

Babst, M., Katzmann, D.J., Estepa-Sabal, E.J., Meerloo, T., and Emr, S.D. (2002). ESCRT-III: an endosome-associated heterooligomeric protein complex required for mvb sorting. Dev Cell *3*, 271-282.

Buchkovich, N.J., Henne, W.M., Tang, S., and Emr, S.D. (2013). Essential N-terminal insertion motif anchors the ESCRT-III filament during MVB vesicle formation. Dev Cell *27*, 201-214.

Henne, W.M., Buchkovich, N.J., Zhao, Y., and Emr, S.D. (2012). The endosomal sorting complex ESCRT-II mediates the assembly and architecture of ESCRT-III helices. Cell *151*, 356-371.

Hierro, A., Sun, J., Rusnak, A.S., Kim, J., Prag, G., Emr, S.D., and Hurley, J.H. (2004). Structure of the ESCRT-II endosomal trafficking complex. Nature *431*, 221-225.

Odorizzi, G., Babst, M., and Emr, S.D. (1998). Fab1p PtdIns(3)P 5-Kinase Function Essential for Protein Sorting in the Multivesicular Body. Cell *95*, 847-858.

Odorizzi, G., Katzmann, D.J., Babst, M., Audhya, A., and Emr, S.D. (2003). Bro1 is an endosome-associated protein that functions in the MVB pathway in Saccharomyces cerevisiae. J Cell Sci *116*, 1893-1903.

Robinson, J., Klionsky, D., Banta, L., and Emr, S. (1988). Protein sorting in Saccharomyces cerevisiae: isolation of mutants defective in the delivery and processing of multiple vacuolar hydrolases. Mol Cell Biol *8(11)*, 4936-4948.

Sato, T.K., Darsow, T., and Emr, S.D. (1998). Vam7p, a SNAP-25-like molecule, and Vam3p, a syntaxin homolog, function together in yeast vacuolar protein trafficking. Mol Cell Biol *18*, 5308-5319.

Sikorski, R.S., and Hieter, P. (1989). A system of shuttle vectors and yeast host strains designed for efficient manipulation of DNA in Saccharomyces cerevisiae. Genetics *22*, 19-27.

Stringer, D.K., and Piper, R.C. (2011). A single ubiquitin is sufficient for cargo protein entry into MVBs in the absence of ESCRT ubiquitination. J Cell Biol *192*, 229-242.

Tang, S., Henne, W.M., Borbat, P.P., Buchkovich, N.J., Freed, J.H., Mao, Y., Fromme, J.C., and Emr, S.D. (2015). Structural basis for activation, assembly and membrane binding of ESCRT-III Snf7 filaments. Elife *4*.

Teis, D., Saksena, S., and Emr, S.D. (2008). Ordered assembly of the ESCRT-III complex on endosomes is required to sequester cargo during MVB formation. Dev Cell *15*, 578-589.
